# An Automated Pipeline for the Analysis of PET Data on the Cortical Surface

**DOI:** 10.3389/fninf.2018.00094

**Published:** 2018-12-10

**Authors:** Arnaud Marcoux, Ninon Burgos, Anne Bertrand, Marc Teichmann, Alexandre Routier, Junhao Wen, Jorge Samper-González, Simona Bottani, Stanley Durrleman, Marie-Odile Habert, Olivier Colliot, for the Alzheimer's Disease Neuroimaging Initiative

**Affiliations:** ^1^Institut du Cerveau et de la Moelle épinière, ICM, Paris, France; ^2^Inserm, U 1127, Paris, France; ^3^CNRS, UMR 7225, Paris, France; ^4^Sorbonne Université, Paris, France; ^5^Inria, Aramis Project-Team, Paris, France; ^6^AP-HP, Departments of Neuroradiology and Neurology, Pitié-Salpétriére Hospital, Paris, France; ^7^Institut du Cerveau et de la Moelle épinière, ICM, FrontLab, Paris, France; ^8^Department of Neurology, National Reference Center for “PPA and rare dementias”, Institute for Memory and Alzheimer's Disease, Pitié Salpêtrière Hospital, AP-HP, Paris, France; ^9^AP-HP, Hôpital Pitié-Salpêtrière, Department of Nuclear Medicine, Paris, France; ^10^Laboratoire d'Imagerie Biomédicale, Sorbonne Universités, UPMC Univ Paris 06, Inserm U 1146, CNRS UMR 7371, Paris, France; ^11^Centre Acquisition et Traitement des Images, Paris, France

**Keywords:** surface analysis, positron emission tomography, PET, brain, neurodegenerative diseases, workflow, pipeline

## Abstract

We present a fully automatic pipeline for the analysis of PET data on the cortical surface. Our pipeline combines tools from FreeSurfer and PETPVC, and consists of (i) co-registration of PET and T1-w MRI (T1) images, (ii) intensity normalization, (iii) partial volume correction, (iv) robust projection of the PET signal onto the subject's cortical surface, (v) spatial normalization to a template, and (vi) atlas statistics. We evaluated the performance of the proposed workflow by performing group comparisons and showed that the approach was able to identify the areas of hypometabolism characteristic of different dementia syndromes: Alzheimer's disease (AD) and both the semantic and logopenic variants of primary progressive aphasia. We also showed that these results were comparable to those obtained with a standard volume-based approach. We then performed individual classifications and showed that vertices can be used as features to differentiate cognitively normal and AD subjects. This pipeline is integrated into Clinica, an open-source software platform for neuroscience studies available at www.clinica.run.

## Introduction

Positron emission tomography (PET) is widely used for the study of neurodegenerative diseases (Jagust, 2006). These diseases are known to be intrinsically linked to aggregates of proteins, such as the tau or beta amyloid proteins, which can be imaged using different PET radiotracers. For instance, ^18^F-AV-1451 allows the visualization of the tau protein in the neurofibrillary tangles (Lowe et al., 2016) and ^18^F-AV-45 binds to beta-amyloid plaques (Wong et al., 2010). ^18^F-Fluorodeoxyglucose (FDG) is the most widely used PET radiopharmaceutical (Hess et al., 2014). As it behaves as an analog of glucose, FDG acts as an indirect marker of synaptic dysfunction (Silverman et al., 2001; Frisoni et al., 2017; Garibotto et al., 2017; Nobili et al., 2018).

Magnetic resonance imaging (MRI) is another modality that plays an important role when studying neurodegenerative diseases (Jagust, 2006). Surface-based analysis has been widely used to study properties of the cortex, such as cortical thickness, based on structural MR images (Fischl and Dale, 2000; Jones et al., 2000; Tustison et al., 2014). This type of analysis emerged after the development of surface extraction approaches (MacDonald et al., 1994; Dale et al., 1999; Fischl et al., 1999a). As an important part of the metabolic activity is located within the cortex, surface-based analyses would be well suited to analyze FDG PET images and allow the joint examination of cortical thickness and hypometabolism on the surface.

Surface-based analysis of FDG PET data can be tracked back to Minoshima et al. (1995b), where the authors used three-dimensional stereotactic surface projection to display the patient's metabolism. This approach is still used in the clinic nowadays, as it gives clinicians an alternative representation of the activity. More sophisticated methods have since been published (Park et al., 2006; Matheson et al., 2017; Vanhoutte et al., 2017; Tan et al., 2018). The surface-based analysis approaches developed by Matheson et al. (2017) and Tan et al. (2018) share the same methodology regarding the representation of the activity: they use the mid surface (surface at equal distance from the pial surface and the white matter/gray matter interface) to directly sample the activity from the coregistered FDG PET image. Only Park et al. (2006) took into account more surfaces (five values for each vertex), drawing a straight line from a vertex on the white surface to the corresponding vertex on the pial surface and sampling at regularly spaced points. Sampling operations on the surface can be performed with the new tools released by FreeSurfer in its 6th version, whose documentation is aggregated in a page called PetSurfer^1^ These new tools comprise partial volume correction algorithms and projection of volume onto surfaces.

The main limitation of the existing methods lies in the difficulty to apply them: the code is usually not publicly available, and when available, the documentation is limited. Here we present a fully automated pipeline for the analysis of PET data on the cortical surface, both in the subject's space and in a common template. This pipeline enables the reproducible surface-based analysis of PET data on large datasets through a non-trivial combination of different tools, mainly from FreeSurfer. It is based on the Brain Imaging Data Structure (BIDS) (Gorgolewski et al., 2016)^2^ for the organization of the input files, which is a standard adopted throughout the neuroimaging community. This pipeline is available within Clinica^3^ (Routier et al., 2018a), an open-source software platform for clinical neuroimaging research studies.

To evaluate the performance of the proposed workflow, we first performed group comparisons. We assessed whether the approach was able to identify the areas of hypometabolism characteristic of different dementia syndromes: Alzheimer's disease (AD) and both the semantic and logopenic variants of primary progressive aphasia. We also compared these results to those obtained with a standard volume-based analysis. We then performed individual classifications to assess whether vertices could be used as features to differentiate cognitively normal (CN) and AD subjects.

## Methods

**Figure 1:**
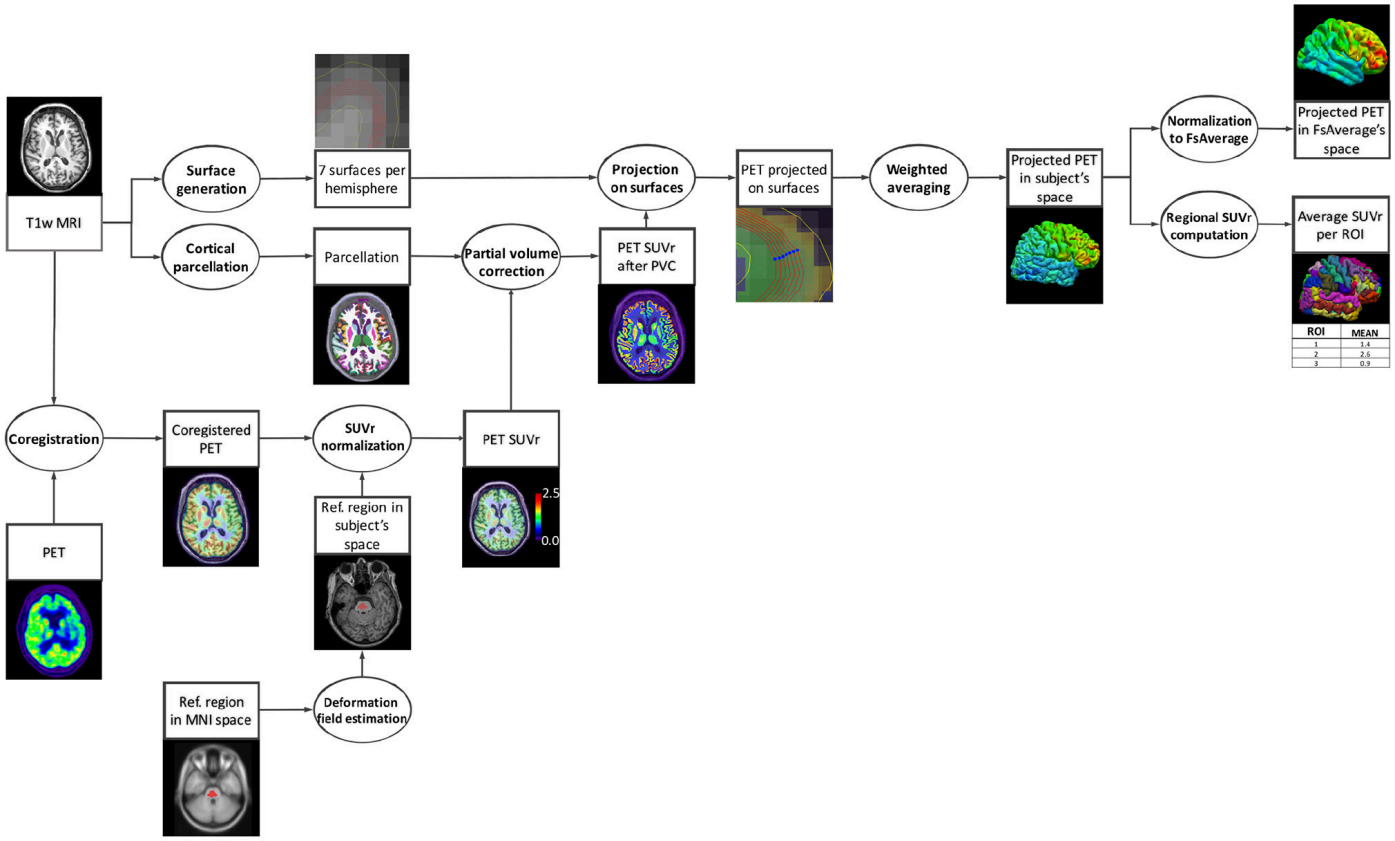
Diagram of the pipeline execution. The subject's T1-w MRI is coregistered with the PET image and the PET image is intensity normalized using the average uptake in a reference region. In parallel, cortical surfaces and a parcellation are generated from the subject's T1-w MRI. The PET image, after partial volume correction performed using the parcellation, is robustly projected onto the cortical surface. Finally, regional mean uptake values are extracted from the projected PET data, and the projected PET signal in the subject's native space is spatially normalized to the standard space of FsAverage.

Our pipeline is composed of six main steps: (i) co-registration of PET and T1-w MR (T1) images, (ii) intensity normalization, (iii) partial volume correction (PVC), (iv) robust projection of the PET signal onto the subject's cortical surface, (v) spatial normalization to a template, and (vi) atlas statistics. A diagram summarizing these steps is displayed in Figure 1.

### Prerequisite

The proposed pipeline requires having run beforehand the *recon-all* pipeline of FreeSurfer (Fischl, 2012). This processing includes segmentation of subcortical structures, extraction of cortical surfaces, cortical thickness estimation, spatial normalization onto the FreeSurfer surface template (FsAverage), and parcellation of cortical regions. Note that a BIDS compliant adaptation of *recon-all* is available in Clinica.

### Coregistration of PET and T1-MRI

The first step of the pipeline consists of rigidly registering the subject's PET image to the T1 image using the tool *spmregister* from FreeSurfer with default parameters.

### Intensity Normalization

To allow for inter-subject comparison, the PET images are then intensity normalized. Standardized uptake value ratios (SUVRs) are generated by dividing the PET images by the mean uptake in a reference region obtained from the Pick atlas in MNI space (Lancaster et al., 1997, 2000; Maldjian et al., 2003). For FDG PET images, the reference region used is the pons (Minoshima et al., 1995a). Registration to MNI space is performed using SPM12. The transformation from the subject's T1 to the MNI template is first estimated, and the inverse deformation is then applied to the reference region. A mask of the pons eroded by a 6-mm sphere is used to ensure that only voxels within the pons are considered when computing the mean uptake.

### Partial Volume Correction

Partial volume correction is performed to limit the spill-out of activity outside of the cortex. The iterative Yang algorithm (Erlandsson et al., 2012) implemented in PETPVC^4^ (Thomas et al., 2016) was chosen for its computational efficiency. Iterative Yang is a volume of interest (VOI) method that assumes that the activity within a region is uniform and thus requires a parcellated T1 image. The *gtmseg* tool from FreeSurfer was used to parcellate the subject's T1 image into 112 regions (comprising 34 cortical gray matter regions per hemisphere). This number was decreased to 50 by fusing certain regions, as suggested by the developer of PETPVC^4^.

### Robust Pet Signal Projection

This step consists of robustly projecting the PET signal onto the subject's cortical surface. First, seven surfaces are generated from the white surface and the cortical thickness (see section Prerequisite) using the *mris_expand* function of FreeSurfer, ranging from 35 to 65% of the cortical thickness with a step *t* = 5%. Since the different surfaces are expanded from the white surface, there is a vertex-to-vertex correspondence between the meshes. For each vertex, the projected PET signal is obtained by computing a weighted average of the PET signal intersecting with the seven surfaces. More weight is given to the surfaces located near the mid distance between the pial and white surfaces as they have a higher probability to be well located within the cortex. We used a normal distribution centered at the mid distance between the pial and white surfaces whose standard deviation was determined so that vertices located on the pial and white surfaces have weights close to zero. This corresponds to the parameters μ = 0.5 and σ = 0.143. The weight given to the surface at x% of the cortical thickness is equal to the integral of the probability density function between $\frac{\left(x-t\right)}{2}$ and $\frac{\left(x+t\right)}{2}$. This results in the following seven weights that we can normalize:

(1) $$\begin{array}{l} \text{C}=[0.0805,\;0.1089,\;0.1306,\;0.1388,\;0.1306,\\ \;0.1089,\;0.0805] \end{array}$$

(2) $${\text{C}}_{\text{normalized},\text{ i}}=\;\frac{{\text{C}}_{\text{i}}}{\sum_{j=1}^{7}{\text{C}}_{j}}$$

(3) $$\begin{array}{l} C_{\text{normalized}}=\;[0.1034,\;0.1399,\;0.1677,\;0.1782,\;0.1677,\\ \;0.1399,\;0.1034]. \end{array}$$

### Registration to a Common Template

To allow group comparison, the cortical surface of each subject is registered to a common template (FsAverage) using the tool *mris_preproc* from FreeSurfer, which performs spherical registration (Fischl et al., 1999b). The PET signal projected onto FsAverage can then be smoothed along the cortical surface using various Gaussian kernels of full width at half maximum (FWHM) of 0, 5, 10, 15, and 20 mm (geodesic distances).

### Atlas Statistics

FreeSurfer, through the *recon-all* pipeline, provides a cortical surface-based parcellation in the subject's space for two atlases: Destrieux and Desikan-Killiany. The mean values of the projected PET signal in each region are computed within our pipeline and stored in a text file. These regional features could be used as inputs for subsequent machine learning or statistical analyses.

### Implementation

**Figure 2:**
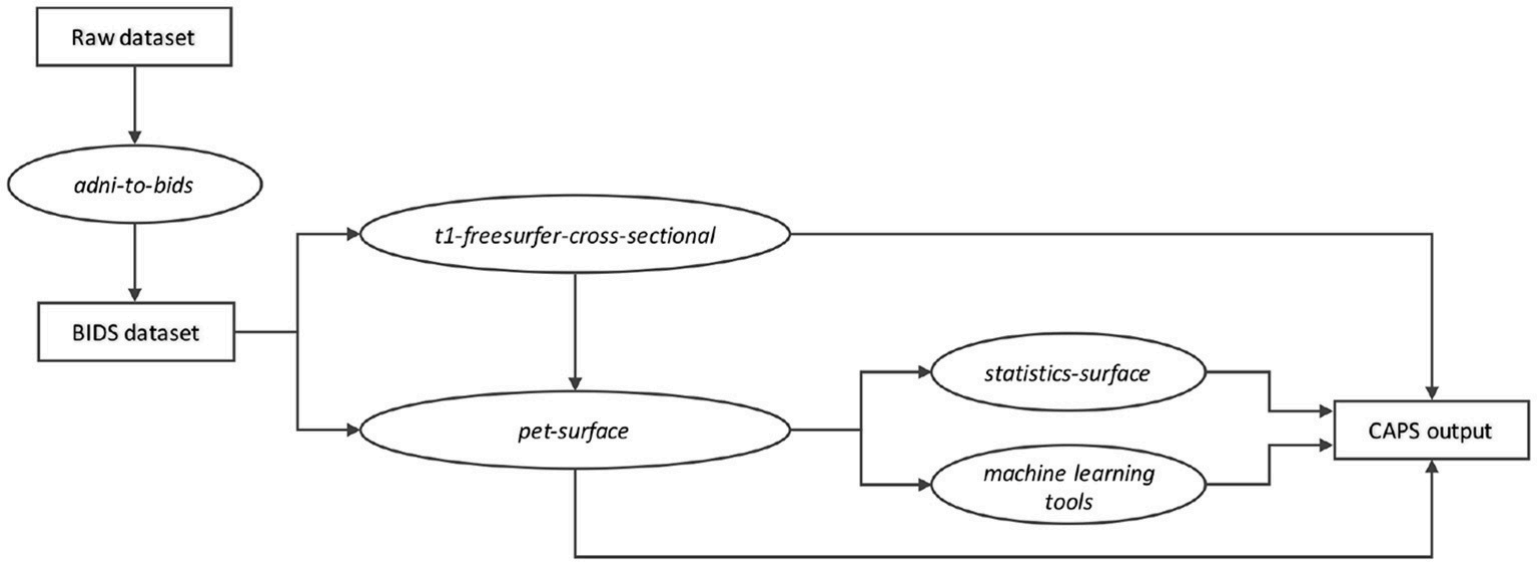
Diagram of the Clinica modules involved in the processing and analysis of the data presented in this paper. The raw dataset is **first** converted into a BIDS compliant format (e.g., using the *adni-to-bids* converter). The *t1-freesurfer-cross-sectional* pipeline (wrapping the *recon-all* command of FreeSurfer) provides the cortical surfaces and the parcellation necessary to run the proposed *pet-surface* pipeline. The PET uptake projected onto the cortical surface can be used to perform individual classification (machine learning module) or group comparison (*statistics-surface* pipeline). All these modules store their outputs in a ClinicA Processed Structure (CAPS) hierarchy. All the names in italics refer to actual command lines of Clinica.

The proposed *pet-surface* pipeline is part of Clinica, a software that aims at making clinical neuroscience easier and more reproducible (Routier et al., 2018a). Clinica contains three main parts. (1) Feature extraction pipelines for different neuroimaging modalities (currently T1-weighted MRI, diffusion MRI, and PET). The pipelines are written in Python, based on the Nipype library (Gorgolewski et al., 2011), and combine different software packages such as FreeSurfer, SPM, FSL, or PETPVC. (2) Statistics and basic machine learning tools that take the different types of features as input. (3) Tools for dataset management as well as tools to curate publicly available datasets such as ADNI and convert them into the BIDS standard (Samper-González et al., 2017, 2018). Clinica uses the BIDS standard for inputs and the outputs of the different pipelines are stored under a specific BIDS-inspired structure called CAPS (ClinicA Processed Structure). A diagram summarizing the different modules of Clinica involved in the processing and analysis of the data presented in this paper is displayed in Figure 2.

## Materials

### Datasets

Part of the data used in the preparation of this article were obtained from the Alzheimer's Disease Neuroimaging Initiative database^5^ The ADNI was launched in 2003 as a public-private partnership, led by Principal Investigator Michael W. Weiner, MD. The primary goal of ADNI has been to test whether serial MRI, PET, other biological markers, and clinical and neuropsychological assessment can be combined to measure the progression of mild cognitive impairment (MCI) and early AD. Over 1,650 participants were recruited across North America during the first three phases of the study (ADNI1, ADNI GO, and ADNI2). Around 400 participants were diagnosed with AD, 900 with MCI and 350 were control subjects. Three main criteria were used to classify the subjects (Petersen et al., 2010). The normal subjects had no memory complaints, while the subjects with MCI and AD both had to have complaints. CN and MCI subjects had a mini-mental state examination (MMSE) score between 24 and 30 (inclusive), and AD subjects between 20 and 26 (inclusive).

Data were also obtained from the CAPP study, a French multicenter investigation on primary progressive aphasias (“PHRC-CAPP”). The different variants of PPA were established by expert neurologists following the international diagnostic criteria of Gorno-Tempini et al. (2011). PPA patients were at an early stage of the disease as reflected by aphasia severity scores ≥3 (normal = 5) in the Boston Diagnostic Aphasia Examination (Mazaux and Orgogozo, 1982). Patients did not present any neurological/psychiatric disease other than PPA. All participants were native French speakers. The local ethics committee approved the study and informed written consent was obtained from the participants.

### Participants

The first two sets of participants considered in our experiments were extracted from the ADNI dataset. The first dataset, denoted ADNI_ALL_, comprises 242 subjects who were either diagnosed as CN at baseline with negative amyloid status, or as AD at baseline with positive amyloid status, and for whom a T1w-MR and an FDG PET scan, with a known effective resolution (to estimate the point spread function), were available. The amyloid status of the subjects was determined from a PiB or an AV45 PET scan with thresholds of 1.47 and 1.10, respectively (Landau et al., 2013). The second dataset, ADNI_SUBSET_, is a subset of ADNI_ALL_ that comprises 30 CN and 30 AD subjects. Tables 1, 2 summarize the demographics, MMSE and amyloid status of the ADNI_ALL_ and ADNI_SUBSET_ participants, respectively.

**Table 1:** Summary of participant demographics, mini-mental state examination (MMSE), and amyloid status for ADNI_ALL_.

**Diagnosis**	***N***	**Age**	**Gender**	**MMSE**	**Amyloid status**
CN	116	72.2 ± 6.1 [56.2, 89.0]	60M/56F	29.0 ± 1.3 [24, 30]	Aβ-
AD	126	74.1 ± 8.1 [55.1, 90.3]	65M/61F	22.9 ± 2.1 [19, 26]	Aβ+

*Values are presented as mean ± SD [range]. CN, cognitively normal; AD, Alzheimer's disease; F, female; M, male. Age is in years*.

**Table 2:** Summary of participant demographics, mini-mental state examination (MMSE), and amyloid status for ADNI_SUBSET_.

**Diagnosis**	***N***	**Age**	**Gender**	**MMSE**	**Amyloid status**
CN	30	72.2 ± 6.3 [56.2, 83.6]	15M/15F	29.1 ± 1.1 [26, 30]	Aβ-
AD	30	74.7 ± 8.0 [55.9, 86.5]	13M/17F	23.0 ± 1.8 [19, 26]	Aβ+

*Values are presented as mean ± SD [range]. CN, cognitively; AD, Alzheimer's disease; F, female; M, male. Age is in years*.

Forty-One patients with semantic variant PPA (svPPA), Twenty-Six patients with logopenic variant PPA (lvPPA), and Twenty-Two control subjects were extracted from the CAPP database (Table 3). In the following, CAPP_SEMANTIC_ denotes the dataset composed of the svPPA and control subjects, and CAPP_LOGOPENIC_ denotes the dataset composed of the lvPPA and control subjects. More details regarding this cohort can be found in Routier et al. (2018b).

**Table 3:** Summary of participant demographics and mini-mental state examination (MMSE) for CAPP.

**Diagnosis**	***N***	**Age**	**Gender**	**MMSE**
CN	22	66.0 ± 7.6 [55.6, 88.1]	7M/15F	27.5 ± 1.4 [26, 30]
svPPA	41	66.0 ± 7.3 [51.7, 88.1]	20M/21F	24.7 ± 2.6 [20, 29]
lvPPA	26	68.5 ± 5.5 [53.0, 77.0]	15M/11F	24.9 ± 3.7 [14, 29]

*CN, cognitively normal; svPPA, semantic variant primary progressive aphasia; lvPPA, logopenic variant primary progressive aphasia; F, female; M, male. Age is in years*.

### Imaging Data

#### ADNI

The acquisition protocols of the 3D T1-w MR images from ADNI 2 can be found in Jack et al. (2010). When available the images corrected for *gradwarp* and B1-inhomogeneity were used, otherwise the original images were selected. The ADNI FDG PET protocol within ADNI GO/2 consisted of a dynamic acquisition of four 5-min frames, 30 to 60 min after injection (Jagust et al., 2015). Images at different stages of preprocessing (frame averaging, spatial alignment, interpolation to a standard voxel size, and smoothing to a common resolution of 8 mm full width at half maximum) are available for download. The images co-registered and averaged across time frames were selected. The curation of the ADNI database and its conversion to the BIDS format was performed using the *adni-to-bids* converter available within Clinica (Samper-González et al., 2017, 2018).

#### CAPP

MRI acquisition was performed on 3 Tesla or 1.5 Tesla scanners depending on the scanner available in each center. The imaging centers all belong to the harmonized national network of the *Centre d'Acquisition et de Traitement d'Images* (CATI) (Habert et al., 2016; Operto et al., 2016). MRI and FDG PET sequences were harmonized by the CATI in order to minimize differences between centers. T1-weighted images were acquired with a 3D gradient echo sequence (240 × 256 acquired matrix; voxel size = 1.0 × 1.0 × 1.0 mm^3^; inversion time = 900 ms; repetition time 2.30 ms; echo time = 2.98 ms; flip angle = 9°). Brain FDG PET scans were obtained 30 min after injection of 2 MBq/kg of ^18^F-FDG. All PET acquisitions were performed in a single session and consisted of 3 × 5 min frames. Images were reconstructed using a conventional 3D iterative algorithm, with a post-reconstruction filter in a 128 × 128 matrix. Acquisition parameters were harmonized for 12 different scanners. Voxel size range from 2 to 3.27 mm. Attenuation, scatter and random coincidence corrections were integrated in the reconstruction. Algorithms with spread function modeling were discarded, even when available. Finally, frames were realigned, averaged and quality-checked by the CATI (Routier et al., 2018b).

## Validation Design

### Group Comparison

Group comparisons were first performed to assess whether the proposed surface-based analysis approach was able to accurately detect differences between cognitively normal and diseased populations. More precisely, a point-wise, vertex-to-vertex model based on the Matlab *SurfStat* toolbox^6^ was used to conduct a group comparison in the whole brain. As all our subjects are in the space of the FsAverage template, we can infer a vertex to vertex comparison using data smoothed with a Gaussian kernel of 20 mm. The general linear model was used to control for the effects of age and sex. Statistics were corrected for multiple comparisons using the random field theory for non-isotropic images (Worsley et al., 1999). A statistical threshold of *p* = 0.05 (at the vertex level) was applied. This whole analysis was performed with the *statistics-surface* pipeline available in Clinica.

Group comparison results obtained with the proposed surface-based approach were then compared to the results obtained with the following volume-based approach. A group template is created using DARTEL, an algorithm for diffeomorphic image registration (Ashburner, 2007), from the subjects' tissue probability maps obtained with SPM12. For each subject, the deformation field from the native MR space to the group template in MNI is then applied to the co-registered PET image. The PET image in MNI space is then intensity normalized using the average activity in a reference region (eroded pons for FDG PET) to generate an SUVR map. The resulting PET images in the group template space are smoothed across the volume with an 8 mm Gaussian kernel. Partial volume correction is not applied, because the aim is to compare our approach to a simple and standard preprocessing strategy (the impact of PVC on the volume-based approach is detailed in the Supplementary Materials). These different steps are implemented in the *t1-volume-new-template* and *pet-volume* pipelines of Clinica [more details can be found in Samper-González et al. (2018)]. These preprocessing steps are consistent with what is commonly done (Ishii et al., 2001; Chételat et al., 2008; Kalpouzos et al., 2009; Gray et al., 2013; Madhavan et al., 2013; Ewers et al., 2014). We then carry out a two-sample *t*-test with age and sex as covariates.

To ease the comparison of the surface-based and volume-based analyses, we normalize the results of the volume-based group comparison to the space of FsAverage. The map of *t*-values obtained with SPM is converted to corrected *p*-values, then mapped from the DARTEL template in MNI to the space of FsAverage and projected onto its cortical mid surface. We compute the Sørensen-Dice coefficient to quantify the overlap of statistically significant difference areas between the surface-based and the voxel-based approach (subsequently projected onto the surface). To do so, the *p*-value maps were binarized using the same threshold of *p* = 0.05 corrected for multiple comparisons.

We also wanted to assess whether our method led to an inflation of false positives. To do so, we followed a procedure using repeated random splits of healthy controls. We performed 1,000 random drawings of 60 subjects among our 116 healthy controls (from ADNI_ALL_). For each drawing, the 60 subjects were assigned to two groups of 30 which were compared. We ensured that the two groups of 30 subjects did not differ for age and sex (using *t*-test for equal means on age and χ^2^ homogeneity test on sex repartition). We then performed a comparison between the two groups of 30 controls, using the same procedure used to compare controls and patients in the other experiments (*statistics-surface* pipeline from Clinica). Among the 1,000 drawings, we counted the number of times the null hypothesis was rejected, i.e., the number of times a group comparison gave at least one significantly different vertex (corrected *p* ≤ 0.05). If the type I error rate is correctly controlled, we should find < 50 drawings (5%) for which the null hypothesis is rejected.

### Individual Classification

Individual classification was performed to assess the ability of the proposed surface-based approach to accurately classify CN Aβ- and AD Aβ+ subjects (ADNI_ALL_ dataset). The PET signals projected onto the cortical surface, in the space of FsAverage, were used as features to feed a linear SVM classifier. To compare surface-based and state-of-the-art volume-based analyses, the PET images in the DARTEL space were also used as features and fed to the linear SVM classifier.

The evaluation of the classification performances mainly followed the recent guidelines provided by Varoquaux et al. (2017). Cross-validation (CV), the classical strategy to maintain the independence of the train set (used to fit the model), and the test set (used to evaluate the performances), was performed. The CV procedure included two nested loops: an outer loop evaluating the classification performances and an inner loop used to optimize the hyperparameter C of the SVM. For the outer loop, we used 250 stratified shuffle splits with a test size of 30%. Note that the splits were kept the same between the voxel and vertex-based approaches. We used an inner *k*-fold with *k* = 10. For each split, the model with the highest balanced accuracy is selected, and the selected models are averaged across splits to benefit of model averaging. We report the full distribution of the balanced accuracy in addition to the mean and empirical standard-deviation. This individual classification was performed with classes implemented in Clinica for the reading of outputs, the linear SVM itself (based on *scikit-learn*), and the cross-validation procedure (Samper-González et al., 2018).

## Results

**Figure 3:**
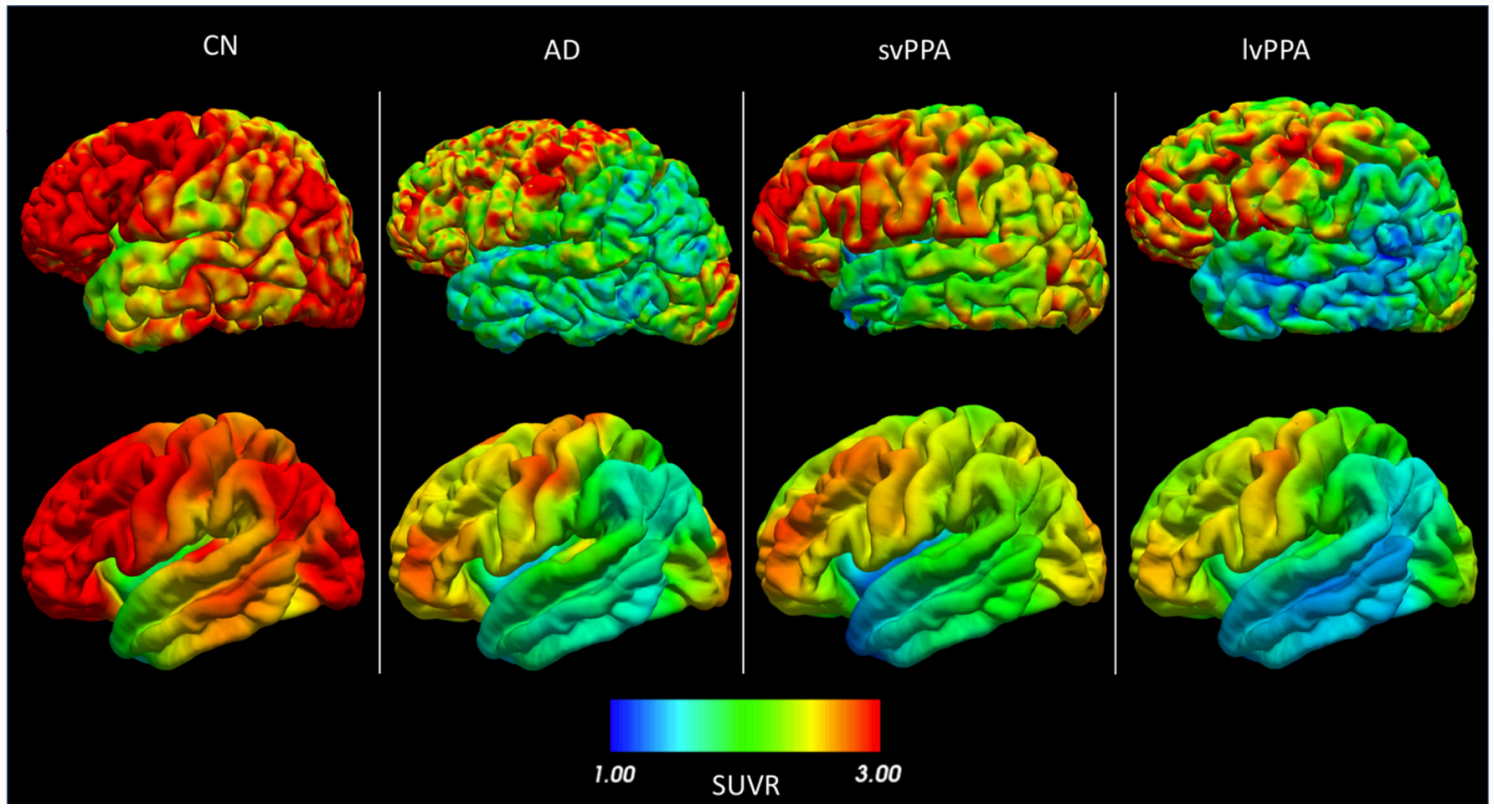
FDG PET SUVR projected onto the cortical surface (left hemisphere) for (from left to right) a cognitively normal subject (CN), a patient with Alzheimer's disease (AD), a patient with semantic variant primary progressive aphasia (svPPA), and a patient with logopenic variant primary progressive aphasia (lvPPA). The first row is the projection in the subject's space. The second row is the same signal for each subject, but warped to FsAverage after smoothing with a 20 mm Gaussian kernel.

The pipeline outputs can be used to display the map of the cortical activity in both the subject's own space and in the common template FsAverage (Figure 3). We note a reduced metabolism for the subject with AD compared to the CN subject, mainly in the temporal and parietal lobes. A strong metabolism reduction localized in the temporal lobe is observed for the patient with svPPA, along with the shrinking of the cortical surface. We observe a reduced activity in both the temporal and parietal lobes for the patient with lvPPA. All these results are consistent with the literature (Herholz et al., 2002; Rabinovici et al., 2008).

### Group Comparison

**Figure 4:**
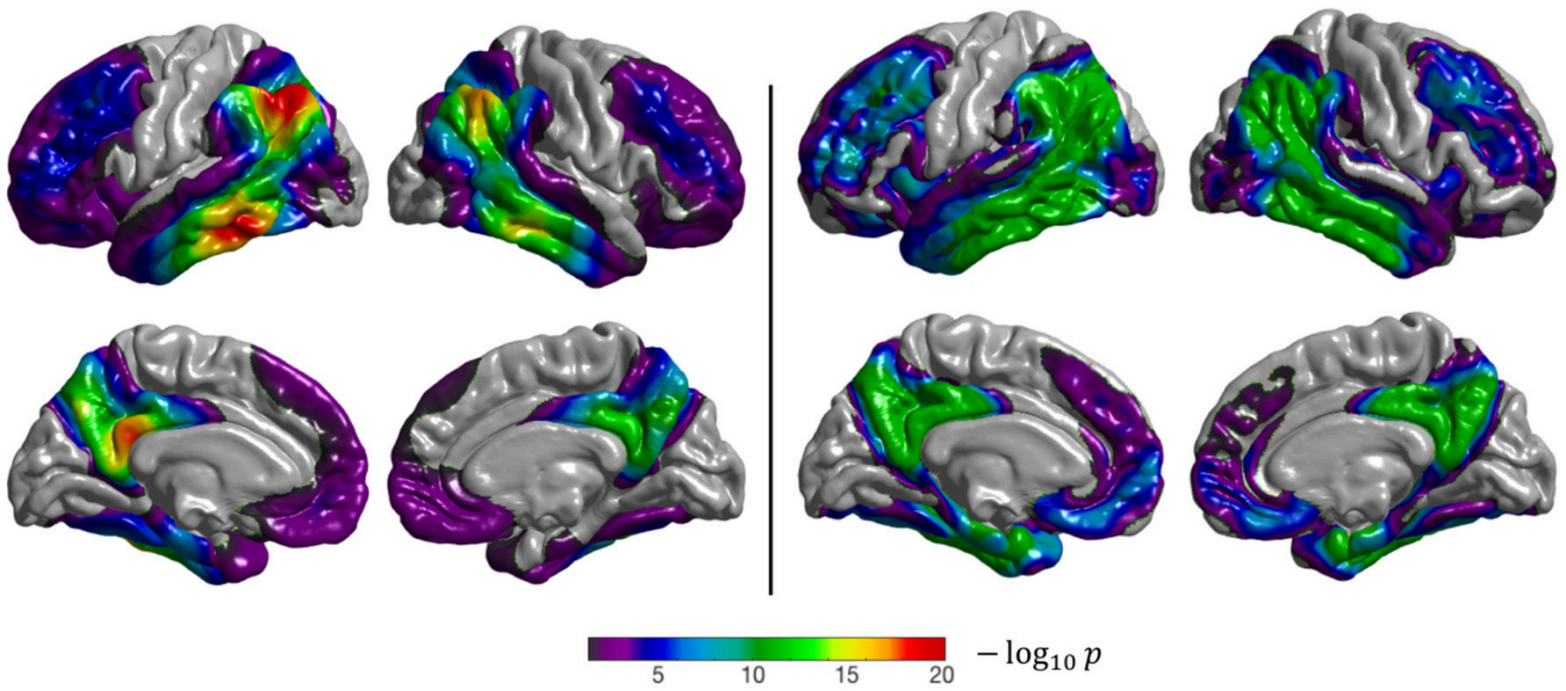
Results of the group comparison (2-sample *t*-test) performed on the ADNI_ALL_ dataset (CN Aβ- vs. AD Aβ+). **Left**: surface-based analysis, **right**: volume-based analysis after projection of the results onto FsAverage. The *p*-values were corrected to the vertex (**left**), and to the voxel (**right**) level, then thresholded to only show significant *p*-values (*p* < 0.05). The Sørensen-Dice coefficient between the two binarized statistical maps is 0.89.

When performing group comparison on the ADNI_ALL_ dataset, the temporal lobe (especially the medial and inferior temporal gyri), posterior cingulate cortex, precuneus, and parietal and frontal lobes show statistically significant differences between CN and AD subjects (Figure 4), as expected (Herholz et al., 2002). Results obtained with the voxel-based analysis projected onto the cortical surface are consistent with those of our method: the same regions are identified even though their appearance is less homogeneous when compared to the surface-based approach. The fact that both approaches identify the same regions shows that the proposed surface-based approach is able to accurately detect differences between CN and AD subjects in a large dataset.

**Figure 5:**
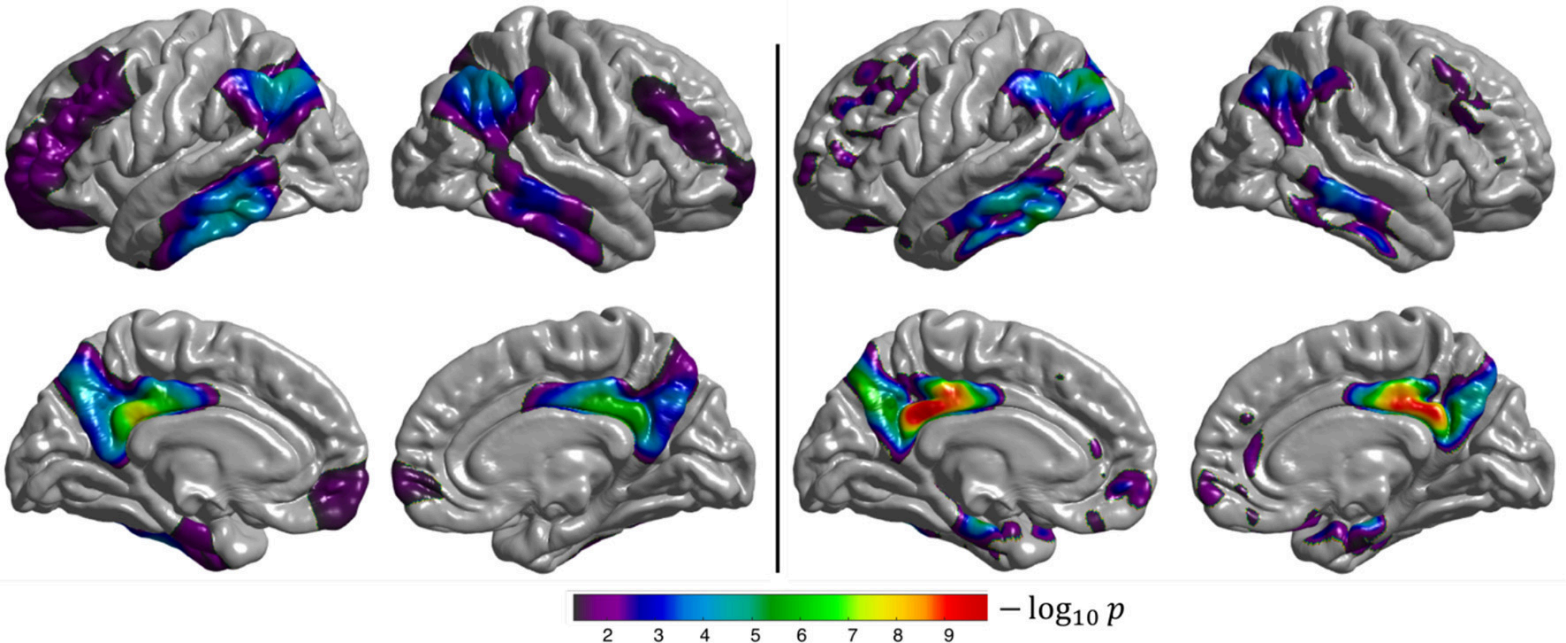
Results of the group comparison (2-sample *t*-test) performed on the ADNI_SUBSET_ dataset (CN Aβ- vs. AD Aβ+). **Left**: surface-based analysis, **right**: volume-based analysis after projection of the results onto FsAverage. The *p*-values were corrected to the vertex (**left**), and to the voxel (**right**) level, then thresholded to only show significant *p*-values (*p* < 0.05). The Sørensen-Dice coefficient between the two binarized statistical maps is 0.71.

When performing group comparison on the ADNI_SUBSET_ dataset (Figure 5), similar (but less extended) regions showed statistically significant differences compared to analysis of the ADNI_ALL_ dataset. This shows that the proposed approach is able to correctly identify differences even in small datasets. Note that the smaller number of subjects compared to ADNI_ALL_ leads to larger *p*-values and less extensive areas.

**Figure 6:**
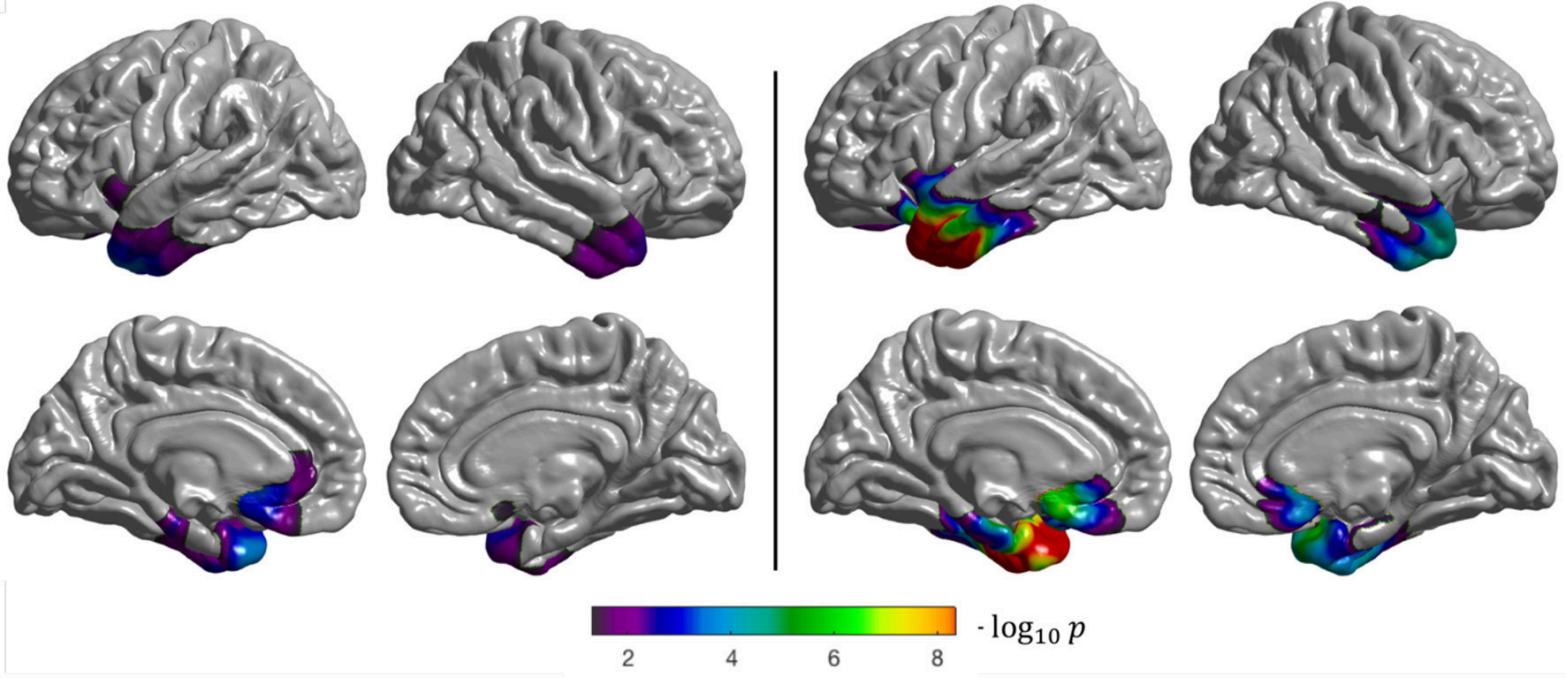
Results of the group comparison (2-sample *t*-test) performed on the CAPP_SEMANTIC_ dataset (CN vs. svPPA). **Left**: surface-based analysis, **right**: volume-based analysis after projection of the results onto FsAverage. The *p*-values were corrected to the vertex (**left**), and to the voxel (**right**) level, then thresholded to only show significant *p*-values (*p* < 0.05). The Sørensen-Dice coefficient between the two binarized statistical maps is 0.59.

The semantic variant of PPA is characterized by hypometabolism focused in the bilateral temporal lobes with an emphasis in the left hemisphere (Jung et al., 2013), which is what we observe from both the surface-based and volume-based group comparisons (Figure 6). However, we note that the regions with statistically significant differences between CN and svPPA subjects are smaller for the surface-based than for the volume-based approach. This might be due to the severe atrophy of the temporal lobe, characteristic of svPPA, which hinders the extraction of the cortical surfaces for some patients, leading to an inaccurate projection of the FDG PET activity, and which may also impact the spherical registration accuracy. This result is also reflected in the low Sørensen-Dice coefficient computed on the thresholded binary maps (0.59).

**Figure 7:**
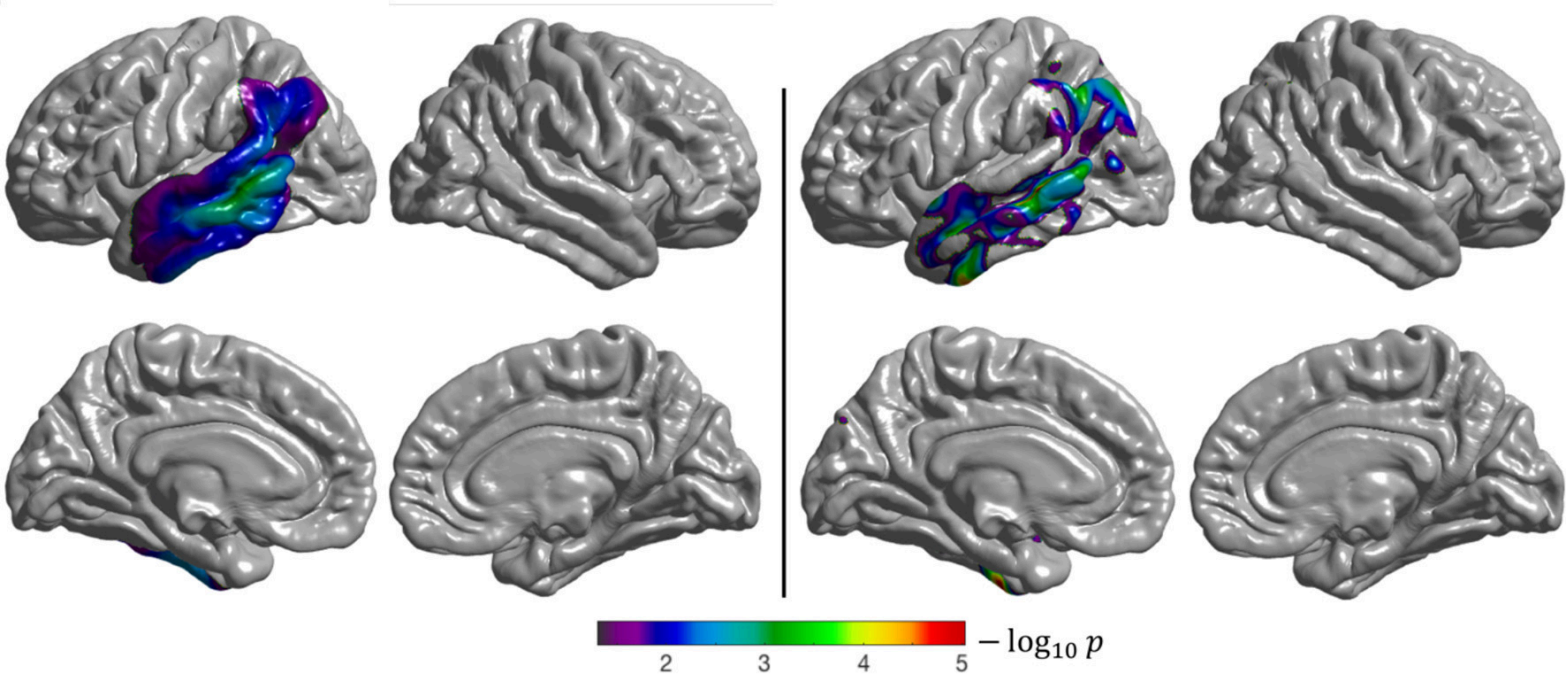
Results of the group comparison (2-sample *t*-test) performed on the CAPP_LOGOPENIC_ dataset (CN vs. lvPPA). **Left**: surface-based analysis, **right**: volume-based analysis after projection of the results onto FsAverage. The *p*-values were corrected to the vertex (**left**), and to the voxel (**right**) level, then thresholded to only show significant *p*-values (*p* < 0.05). The Sørensen-dice coefficient between the **two** binarized statistical maps is 0.71.

Both the surface-based and volume-based group comparisons identified the temporal and parietal lobes as being statistically significantly different between CN and lvPPA subjects (Figure 7). These results are consistent with the findings presented in Henry and Gorno-Tempini (2010). The discontinuity observed in the statistical difference map obtained with the volume-based approach, whose results are projected onto the cortical surface, can be explained by the fact that FDG PET images are smoothed across the volume in MNI space, and not along the surface as for the surface-based analysis. This means that the resulting *t*-statistic volume map obtained with SPM does not always intersect with the cortical surface, leading to “holes” when projected onto FsAverage. Nevertheless, patterns of statistical differences are consistent between the two approaches.

Regarding the assessment of the number of false positives performed on the CN subjects from ADNI_ALL_, we found that it exists (at least one) statistically different vertex in 4.2% of the group comparisons computed. This value reinforces our belief that our method does not create spurious differences, being less than our 5% threshold of significance.

### Individual Classification

**Figure 8:**
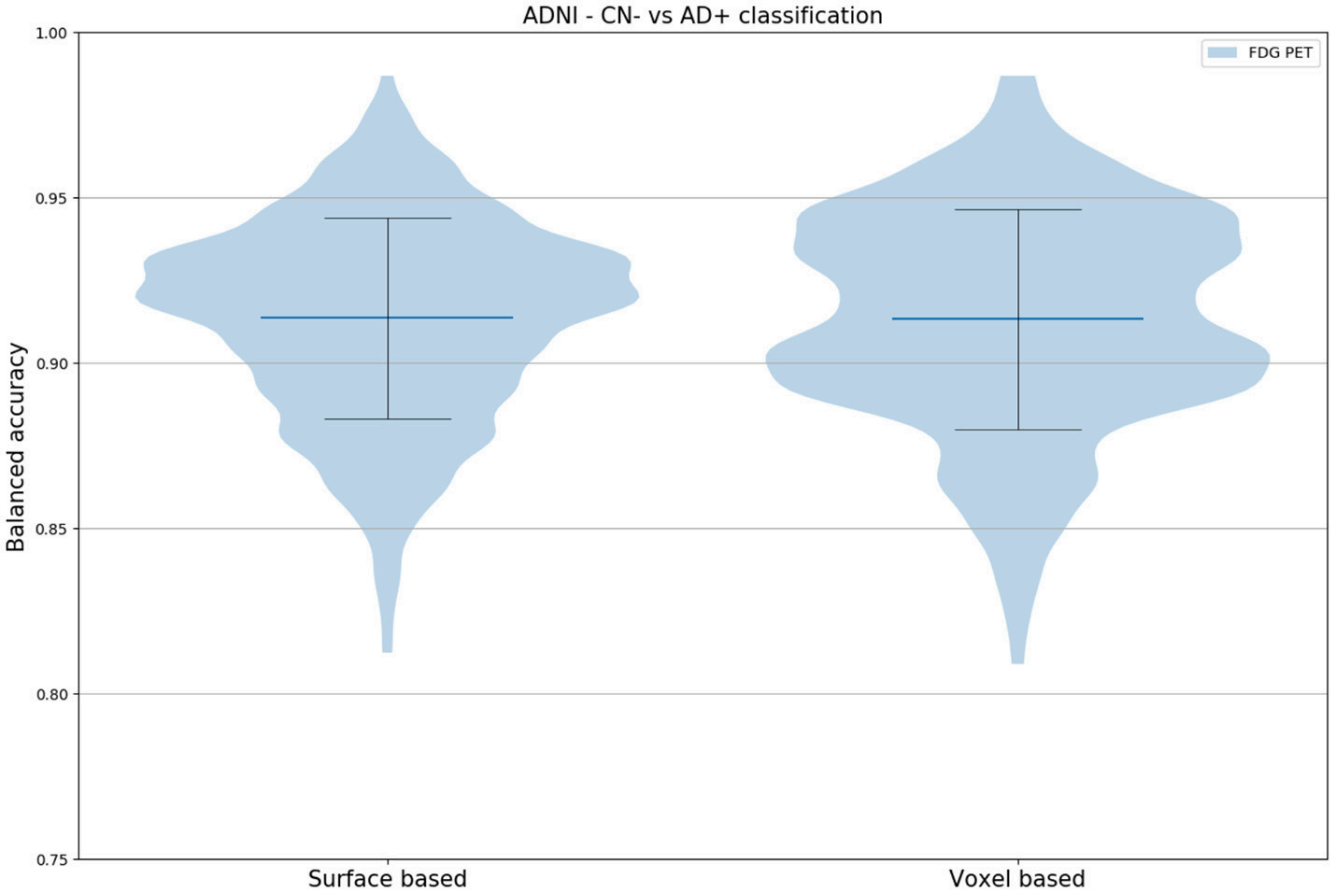
Distribution of the balanced accuracy across 250 runs obtained when classifying CN Aß- vs. AD Aß+ subjects (ADNI_ALL_ dataset) using a linear SVM fed with either vertices (obtained with the proposed method) or voxels. In both cases, no smoothing was applied.

Linear SVM classification of (surface-based) vertex features led to a mean balanced accuracy of 91.36% with a standard deviation of 3.03% (Figure 8). The same classification approach but with voxel features [as done in Samper-González et al. (2018), for the same task and using the same dataset] led to a mean balanced accuracy of 91.33% with a standard deviation of 3.33%. These results show that the proposed surface-based approach is able to provide as accurate classification results as a volume-based approach.

We assessed the proportion of subjects for whom the two classification results agreed. As mentioned in section Individual Classification, the linear SVM were trained and tested on the same 250 stratified shuffle splits for the vertex features and the voxel features. For each of the 250 splits, 70% of the subject list is randomly affected to the training set, and 30% to the testing set. Thus, for each split, 30% of 242 subjects are classified. In total, 0.3 × 242 subjects × 250 runs = 18,150 individual classifications are performed with the surface and with the volume-based linear SVM. We obtained the same prediction between vertex or voxel features in 92.64% of the cases. This shows that the classifiers are in strong agreement and that correctly/incorrectly classified subjects are the same in the vast majority of cases. The most relevant features involved in vertex- and voxel-based individual classifications are displayed in the Supplementary Materials.

## Discussion and Conclusion

We presented a fully automatic pipeline that enables the analysis of PET data on the cortical surface. We evaluated its performance by performing group comparison and individual classification for two different diseases (AD and PPA). This evaluation demonstrated that the developed pipeline is able to identify the areas of hypometabolism characteristic of AD and of the semantic and logopenic variants of PPA, and to individually differentiate CN and AD subjects. We also showed that this method does not create spurious differences.

The proposed surface-based analysis approach relies on tools from different software packages, mainly FreeSurfer and PETPVC, which are combined to build a pipeline able to automatically perform the pre-processing and projection of PET data on the cortical surface. This pipeline is part of the software platform Clinica, under the name *pet-surface*. The target audience of Clinica is mainly of two types. First, scientists or clinicians conducting clinical neuroscience studies involving multimodal imaging, typically not experts in image processing for all of the involved imaging modalities or in statistical analysis. Second, researchers developing advanced machine learning algorithms, typically not experts in brain image analysis. The main objective of Clinica is to enable reproducible research. This is done by developing pipelines performing data processing and analysis in a standardized way, and that can be run thanks to simple command lines. A downside of this approach is its limited customization options. In particular, it is not possible to specify a different reference region for the PET intensity normalization. However, this option could easily be added to future releases if the need arises. Note that the vast majority of the operations presented in this study was conducted with Clinica (see section Validation design). Because Clinica relies on Nipype to build pipelines, the processing can be parallelized over subjects, reducing the total time needed for the computation. Running *pet-surface* for a subject takes approximately 1 h on an Intel® Core™ i7-4870HQ CPU @ 2.50GHz.

We mainly rely on the *mris_expand* function of FreeSurfer rather than on *vol2surf*. The *vol2surf* function allows the user to project volume data onto the cortical surface by sampling along the normal of the white surface, by steps corresponding to fractions of the cortical thickness. We observed that the main issue with this approach is its lack of accuracy and robustness, as there is no constraint to ensure that the last sample will be on the corresponding vertex of the pial surface. Another solution proposed by FreeSurfer is to use the normal from the mid surface, starting on the side of the white surface and going toward the pial surface, sampling by steps proportional to the thickness. Here again we observed that, because of different folding patterns, sampling points could be located outside the cortical ribbon. Our approach, using deformable surfaces obtained with *mris_expand*, ensures that the seven surfaces that are used to project the signal follow the folding patterns and stay within the cortical ribbon. This is further ensured by the proposed weighting scheme, which gives more weight to surfaces located near the mid distance between pial and white surfaces. Note that we do not claim that this weighting scheme is optimal and other schemes could be considered.

We selected Iterative Yang (Erlandsson et al., 2012), a state-of-the-art volume-based method, to perform partial volume correction. This method might not be the best suited to identify small hypometabolic areas (Hutton et al., 2013) but, as our approach is oriented toward group analysis, we favor a method providing high recovery coefficient. Surface-based approaches such as the one developed by Funck et al. (2014) could be an alternative to detect small uptake variations.

Individual classification of patients with Alzheimer's disease vs. cognitively normal subjects demonstrated promising results on a large dataset, obtaining the same balanced accuracy as a standard volume-based approach (surface-based: 91.36%, volume-based: 91.33%), with a reduced standard deviation across runs (surface-based: 3.03%, volume-based: 3.33%). When performing group comparison on the ADNI_ALL_, ADNI_SUBSET_, and CAPP_LOGOPENIC_ datasets, we showed that the proposed approach was able to detect the expected differences between control and diseased subjects, and that patterns of statistical differences were consistent between the surface-based and a standard volume-based method. However, we observed that the accuracy of the proposed approach can be limited when applied to subjects with severe focal atrophy, such as subjects with svPPA. This is related to the difficulty of extracting surfaces and generating accurate parcellations in these areas. This limitation was not observed for the subjects with AD and lvPPA. Finally, the results of the experiment using repeated random splits of healthy controls showed that the type I error rate is correctly controlled, suggesting that our method does not create spurious differences.

The results of our study show that both surface-based and volume-based approaches can be used to analyze PET data. The main advantage of the proposed approach is that it allows the joint analysis of multiple modalities on the cortical surface, such as cortical thickness and metabolism. Note however that the volume-based analysis is recommended when the signal of interest can be located outside of the cortex (e.g., in the hippocampi) or when severe atrophy is expected. Nevertheless, in the field of aging research we believe that our method is appropriate, as the cortical thinning is not critical. Finally, note that MR image quality (that depends on various factors including field strength and different types of correction) can potentially impact on all image processing pipelines that rely on MRI. For the specific case of cortical thickness, the impact of field strength has been previously studied in Han et al. (2006). This paper showed that mean thickness values are higher at 3T than at 1.5T, the order of magnitude of these differences being around 0.1 mm. We believe that such differences would have a limited impact on PET projection given that: (i) this is very small compared to the resolution of PET data; (ii) our method includes a robust sampling scheme which avoids points which are close to the boundaries (sampling from 35 to 65% of the cortical thickness).

To conclude, we introduced a robust and automatic pipeline to project PET signal onto the cortical surface that can be used both for visualization and data analysis purposes. This approach is particularly interesting for multimodal analyses as data extracted from different modalities can be analyzed in the same way (e.g., cortical thickness from MRI and metabolism from FDG PET). By applying it to a large number of control subjects and patients with AD and two variants of primary progressive aphasia, we showed that the proposed surface-based approach was able to identify areas where hypometabolism was expected when comparing control and diseased subjects, and that these findings were consistent with those of a standard volume-based approach. By performing individual classification tasks on a large dataset of control and AD subjects, we showed that our method performed equally well than a standard voxel-based approach. The proposed pipeline, which is part of the publicly available Clinica platform (under the name *pet-surface*), should enable the joint analysis of PET and MRI data on the cortical surface.

## Author Contributions

AM Guarantor of integrity of entire study. AM, NB Literature research; MT, M-OH Clinical studies; AM Statistical analysis. All authors study concepts/study design or data acquisition or data analysis/interpretation, manuscript drafting or manuscript revision for important intellectual content, approval of final version of submitted manuscript, and manuscript editing.

### Conflict of Interest Statement

Competing financial interests unrelated to the present article: OC reports having received speaker fees from Roche and that his laboratory has received grants (paid to the institution) from Air Liquide Medical Systems, Qynapse and myBrainTechnologies. The remaining authors declare that the research was conducted in the absence of any commercial or financial relationships that could be construed as a potential conflict of interest.

## Footnotes

^1^https://surfer.nmr.mgh.harvard.edu/fswiki/PetSurfer.

^2^https://bids.neuroimaging.io/

^3^www.clinica.run

^4^https://github.com/UCL/PETPVC

^5^http://adni.loni.usc.edu/.

^6^http://www.math.mcgill.ca/keith/surfstat/
