# Supplementary material for: An Automated Pipeline for the Analysis of PET Data on the Cortical Surface
Source: Front Neuroinform. 2018 Dec 10;12:94. doi: 10.3389/fninf.2018.00094 (PMC6296445; doi:10.3389/fninf.2018.00094)
Supplement: Supplementary file 1 [file Data_Sheet_1.docx]

Supplementary Material

**An Automated Pipeline for the Analysis of PET Data on the Cortical Surface­­**

**Arnaud Marcoux^1,2,3,4,5^, Ninon Burgos^1,2,3,4,5^, Anne Bertrand^1,2,3,4,5,6,†^, Marc Teichmann^2,3,4,7,8^, Alexandre Routier^2,3,4,5,7^, Junhao Wen^1,2,3,4,5^, Jorge Samper-González^1,2,3,4,5^, Simona Bottani^1,2,3,4,5^, Stanley Durrleman^1,2,3,4,5^, Marie-Odile Habert^9,10,11^, Olivier Colliot^1,2,3,4,5,6,*^, for the Alzheimer’s Disease Neuroimaging Initiative^[[1]](#footnote-1)^§**

^1^Institut du Cerveau et de la Moelle épinière, ICM, F-75013, Paris, France

^2^Inserm, U 1127, F-75013, Paris, France

^3^CNRS, UMR 7225, F-75013, Paris, France

^4^Sorbonne Université, F-75013, Paris, France

^5^Inria, Aramis project-team, F-75013, Paris, France

^6^AP-HP, Departments of Neuroradiology and Neurology, Pitié-Salpêtrière Hospital, Paris, France

^7^Institut du Cerveau et de la Moelle épinière, ICM, FrontLab, F-75013, Paris, France

^8^Department of Neurology, National Reference Center for "PPA and rare dementias", Institute for Memory and Alzheimer's Disease, Pitié Salpêtrière Hospital, AP-HP, Paris. France

^9^AP-HP, Hôpital Pitié-Salpêtrière, Department of Nuclear Medicine, Paris, F-75013, France

^10^Laboratoire d’Imagerie Biomédicale, Sorbonne Universités, UPMC Univ Paris 06, Inserm U 1146, CNRS UMR 7371, Paris, France

^11^Centre Acquisition et Traitement des Images, Paris, France

**^†^** Deceased, March 2^nd^, 2018

* Corresponding author:

Olivier Colliot

ICM – Brain and Spinal Cord Institute

ARAMIS team

Pitié-Salpêtrière Hospital, 47-83, boulevard de l’Hôpital, 75651 Paris Cedex 13, France

E-mail: [olivier.colliot@upmc.fr](mailto:olivier.colliot@upmc.fr)

# Influence of partial volume correction on group comparison for the voxel based approach

To assess the influence of partial volume correction (PVC) on the volume-based approach (subsequently projected onto the surface), we compared results obtained with and without PVC, using the ADNI_SUBSET_ database. PVC was performed using the same method (iterative Yang) and the same segmentation (*gtmseg* tool from FreeSurfer) as for the surface-based method. The result is presented in Figure 1.


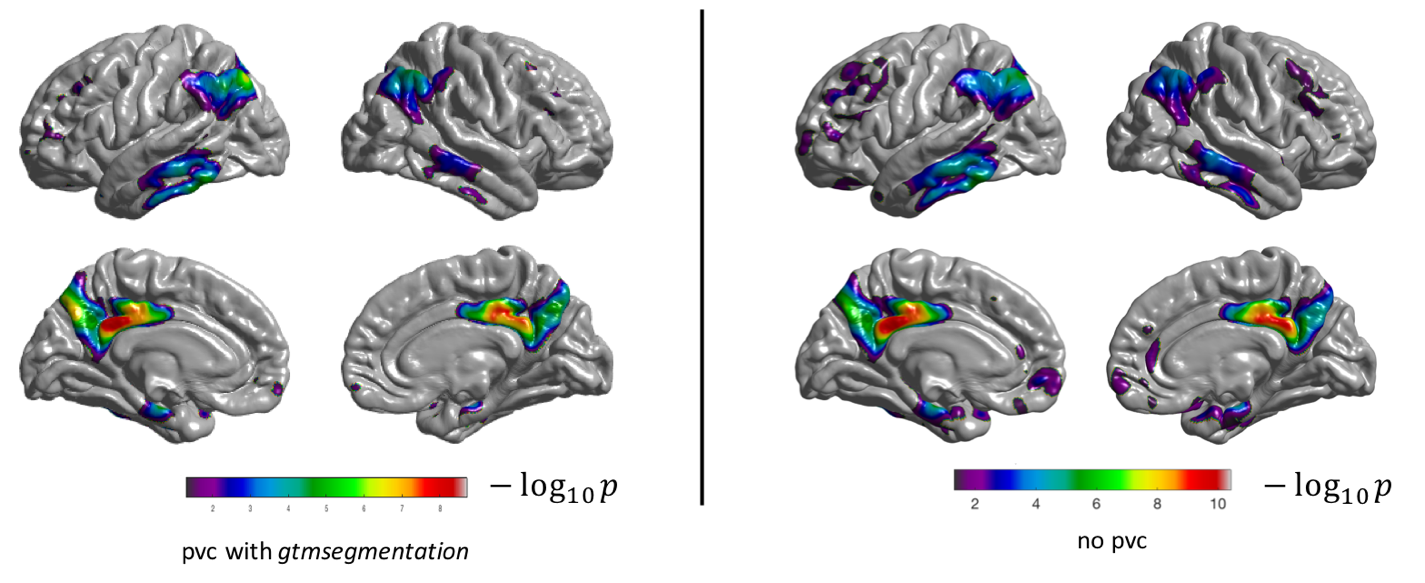


**Figure 1.** Influence of the partial volume correction (PVC) on the volume-based analysis results. Results of the group comparison (2-sample t-test) performed on the ADNI_SUBSET_ dataset. Left: with partial volume correction (with segmentation from gtmseg). Right: without partial volume correction. In both cases, results are projected onto FsAverage. The p-values were corrected to the voxel level, then thresholded to only show significant p-values (p < 0.05).

One can appreciate that PVC had very little impact on the results of the volume-based analysis. We thus decided to not perform PVC for the volume-based analysis.

# Features involved in individual classification task

To visualize the features that contribute the most to the classification of CN Aβ- vs AD Aβ+ for both the vertex- or voxel-based approaches, we studied the corresponding coefficients of the optimal hyperplanes, also called weight maps. The weight map for the surface method is displayed in Figure 2. Note that the input feature maps were not smoothed which results in scattered weights. The weight map for the volume method is displayed in Figure 3. For both methods, the weight maps involve predominantly the temporal lobe, the posterior cingulate cortex, the precuneus as well as other regions of the parietal and frontal lobes, which are regions known to be characteristic of AD.


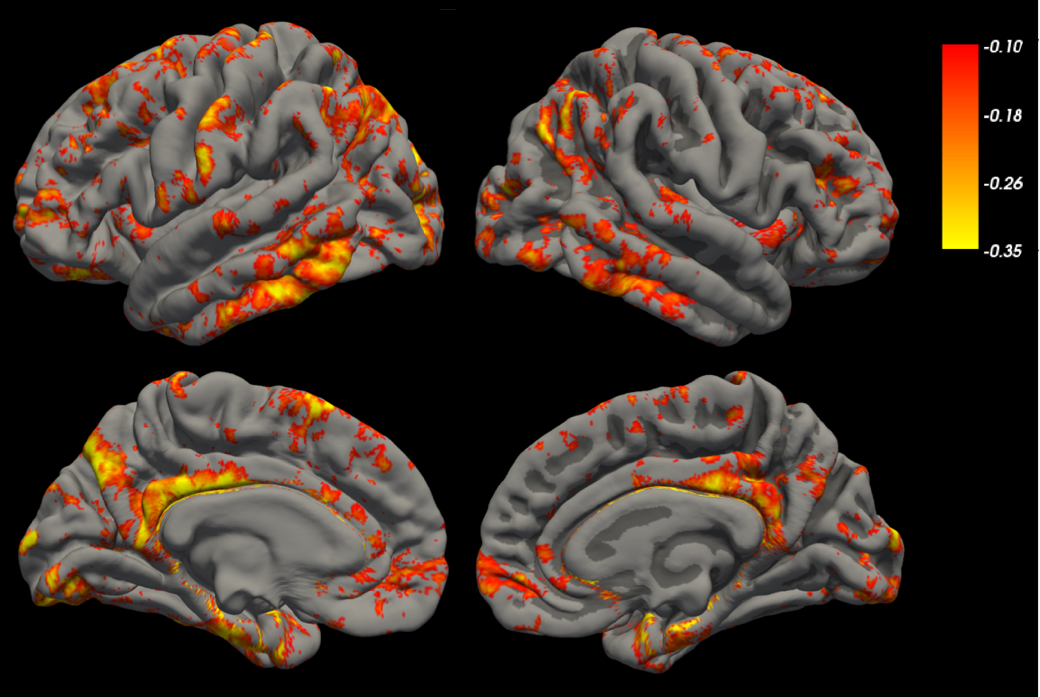


**Figure 2.** Vertices that contribute the most to the classification of CN Aβ- vs AD Aβ+ (ADNI_ALL_ dataset) when using the surface-based approach. Only negative coefficients with an arbitrary threshold are displayed.


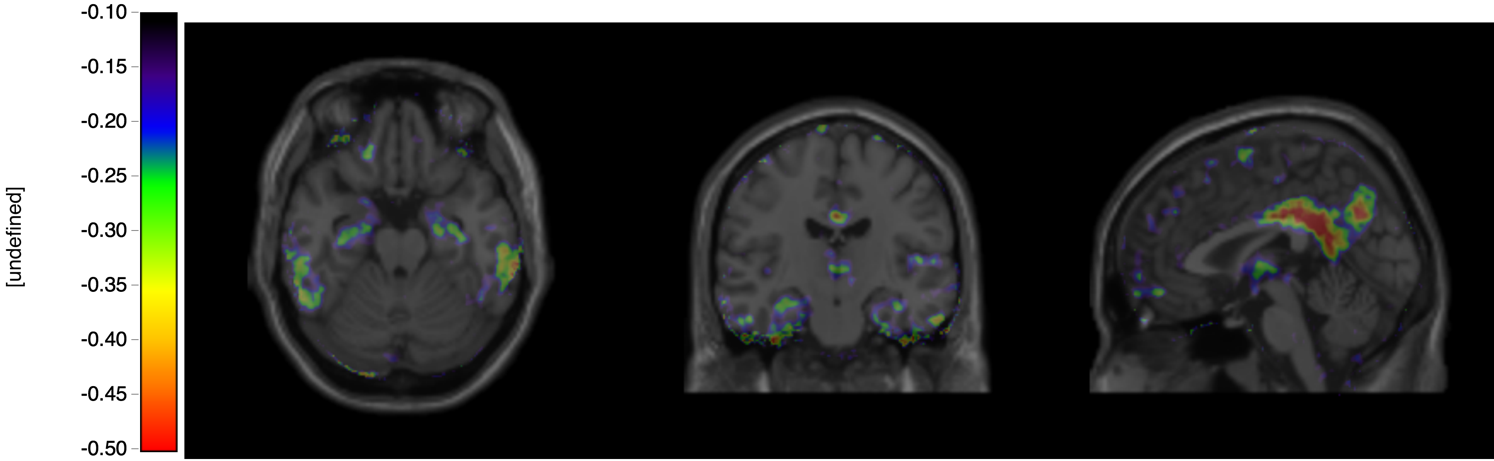


**Figure 3.** Voxels that contribute the most to the classification of CN Aβ- vs AD Aβ+ (ADNI-ALL dataset) when using the volume-based approach. Only negative coefficients with an arbitrary threshold are displayed.

1. § Data used in preparation of this article were obtained from the Alzheimer’s Disease Neuroimaging Initiative (ADNI) database (adni.loni.usc.edu). As such, the investigators within the ADNI contributed to the design and implementation of ADNI and/or provided data but did not participate in analysis or writing of this report. A complete listing of ADNI investigators can be found at: http://adni.loni.usc.edu/wp-content/ uploads/how_to_apply/ADNI_Acknowledgement_List.pdf. [↑](#footnote-ref-1)
